# Preoperative diagnosis of ileal duplication cyst in an adult using dynamic imaging and treatment with single-incision laparoscopic surgery

**DOI:** 10.1093/jscr/rjaf834

**Published:** 2025-10-17

**Authors:** Katsuki Danno, Kenta Takahashi, Masatoshi Nomura, Tadafumi Fukata, Masaya Higashiguchi, Shinya Urakawa, Kozo Noguchi, Takafumi Hirao, Mitsugu Sekimoto, Yoshio Oka

**Affiliations:** Department of Surgery, Minoh City Hospital, 5-7-1 Kayano, Minoh 562-0014, Osaka, Japan; Department of Surgery, Minoh City Hospital, 5-7-1 Kayano, Minoh 562-0014, Osaka, Japan; Department of Surgery, Minoh City Hospital, 5-7-1 Kayano, Minoh 562-0014, Osaka, Japan; Department of Surgery, Minoh City Hospital, 5-7-1 Kayano, Minoh 562-0014, Osaka, Japan; Department of Surgery, Minoh City Hospital, 5-7-1 Kayano, Minoh 562-0014, Osaka, Japan; Department of Surgery, Minoh City Hospital, 5-7-1 Kayano, Minoh 562-0014, Osaka, Japan; Department of Surgery, Minoh City Hospital, 5-7-1 Kayano, Minoh 562-0014, Osaka, Japan; Department of Surgery, Minoh City Hospital, 5-7-1 Kayano, Minoh 562-0014, Osaka, Japan; Department of Surgery, Minoh City Hospital, 5-7-1 Kayano, Minoh 562-0014, Osaka, Japan; Department of Surgery, Minoh City Hospital, 5-7-1 Kayano, Minoh 562-0014, Osaka, Japan

**Keywords:** duplication cyst, adult, ultrasonography, MRI, single-incision laparoscopic surgery

## Abstract

We report a rare case of an ileal duplication cyst diagnosed preoperatively in a 72-year-old woman who presented with incidental hematuria. Imaging studies including abdominal ultrasonography and magnetic resonance imaging (MRI) revealed a 6-cm cystic lesion exhibiting peristaltic movement and layered wall structure, suggestive of an alimentary duplication cyst. Based on these characteristic findings, a single-incision laparoscopic resection of the cyst and adjacent ileum was performed without complications. Histological examination confirmed a non-communicating cystic duplication with smooth muscle and columnar epithelium. The patient recovered uneventfully. This case highlights the diagnostic value of dynamic imaging—particularly ultrasound and MRI—in detecting rare gastrointestinal duplications in adults, and supports minimally invasive resection as a safe and effective treatment strategy.

## Introduction

Alimentary tract duplication is a rare congenital malformation characterized by a smooth muscle-lined cyst or tubular structure that shares a wall with the native gastrointestinal tract [1]. These lesions can occur anywhere from the oral cavity to the rectum, with the ileum being the most commonly affected site [2]. Most cases are diagnosed in infancy or early childhood due to symptoms such as abdominal pain, vomiting, or bowel obstruction [3]. In contrast, adult cases are extremely rare and frequently discovered incidentally during imaging performed for unrelated conditions [4].

Preoperative diagnosis of duplication cysts in adults is challenging, because imaging findings may mimic other cystic lesions such as mesenteric cysts, Meckel’s diverticulum, or adnexal tumors [5]. While traditional imaging modalities such as computed tomography (CT) and standard ultrasonography may demonstrate a cystic mass, they often lack specific features to confirm the diagnosis. Here, we present a rare case of an ileal duplication cyst in an elderly patient, diagnosed preoperatively based on dynamic imaging and treated successfully with single-incision laparoscopic surgery.

## Case presentation

A 72-year-old woman was referred to our hospital for evaluation of transient hematuria. She had no abdominal symptoms, and her physical examination was unremarkable. Laboratory data, including complete blood count, inflammatory markers, and tumour markers (CEA, CA19-9, and AFP), were all within normal limits. Contrast-enhanced abdominal CT did not reveal any urological abnormalities but incidentally identified a 6-cm cystic lesion located on the right side of the uterus.

**Figure 1:**
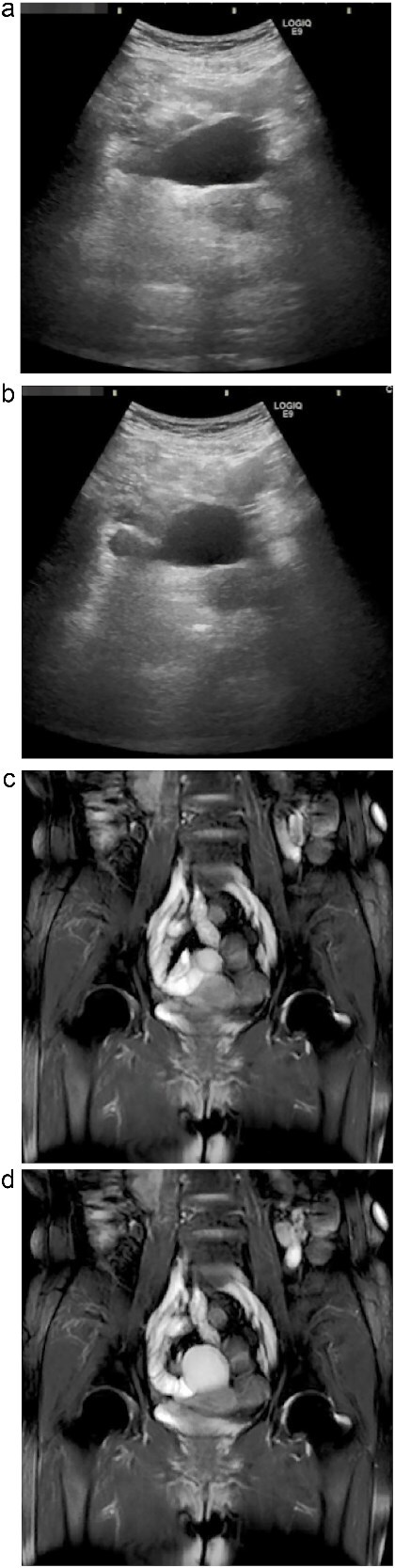
Dynamic ultrasonography and MRI of the abdominal cystic lesion. (a, b) Abdominal ultrasonography revealed a 6-cm cystic lesion with a layered wall structure. Image (b) was taken 4 s after (a), showing deformation of the cyst wall, indicating peristaltic motion. (c, d) Coronal T2-weighted MRI demonstrated a high-intensity cystic lesion adjacent to the ileum. Image (d) was obtained 1.5 s after (c), also showing peristaltic movement of the cyst wall. These dynamic imaging features suggested a duplication cyst with smooth muscle activity.

Subsequent abdominal ultrasonography revealed a well-defined cystic mass with a distinct layered wall. Notably, real-time observation demonstrated peristaltic movement of the lesion over several seconds, suggesting active smooth muscle within the wall (Fig. 1a and b; Supplementary Video 1). Magnetic resonance imaging (MRI) further characterized the lesion as a hyperintense structure on T2-weighted imaging, again demonstrating peristaltic activity on sequential dynamic sequences (Fig 1c and d; Supplementary Video 2). The cyst was closely attached to the ileum but showed no communication with the intestinal lumen.

**Figure 2:**
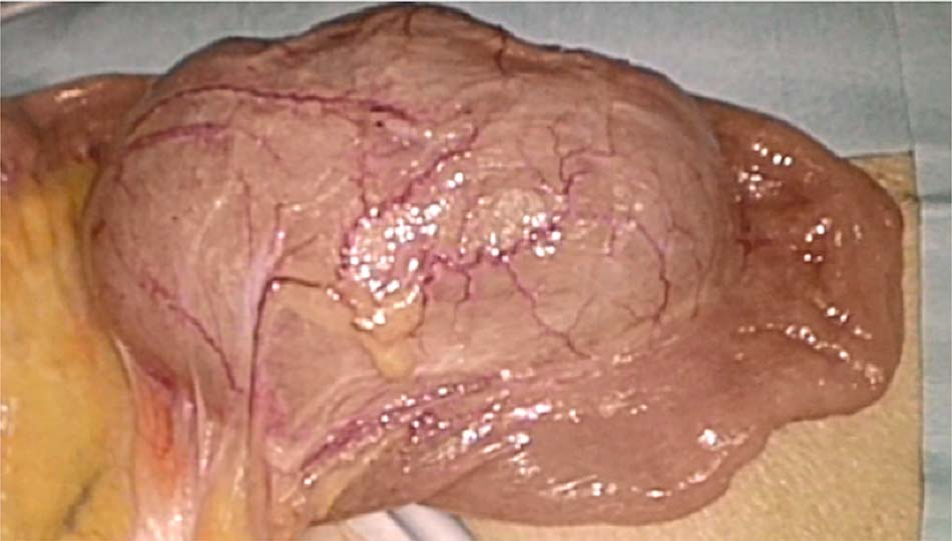
Intraoperative findings during single-incision laparoscopic surgery. The duplication cyst was located on the mesenteric side of the ileum, ~40 cm from the ileocecal valve. The lesion showed visible peristalsis and shared a muscular wall with the adjacent ileum.

Based on these dynamic imaging findings, the lesion was diagnosed preoperatively as an ileal duplication cyst, and surgical resection was planned. A single-incision laparoscopic approach was employed. Intraoperatively, a cystic mass ~7 × 6 cm in size was identified on the mesenteric side of the ileum, ~40 cm proximal to the ileocecal valve. The lesion showed visible peristalsis and shared a muscular wall with the adjacent ileum (Fig. 2). Due to partial adherence to the normal ileum, the cyst and a short segment of ileum were resected en bloc, followed by functional end-to-end anastomosis using a linear stapler.

**Figure 3:**
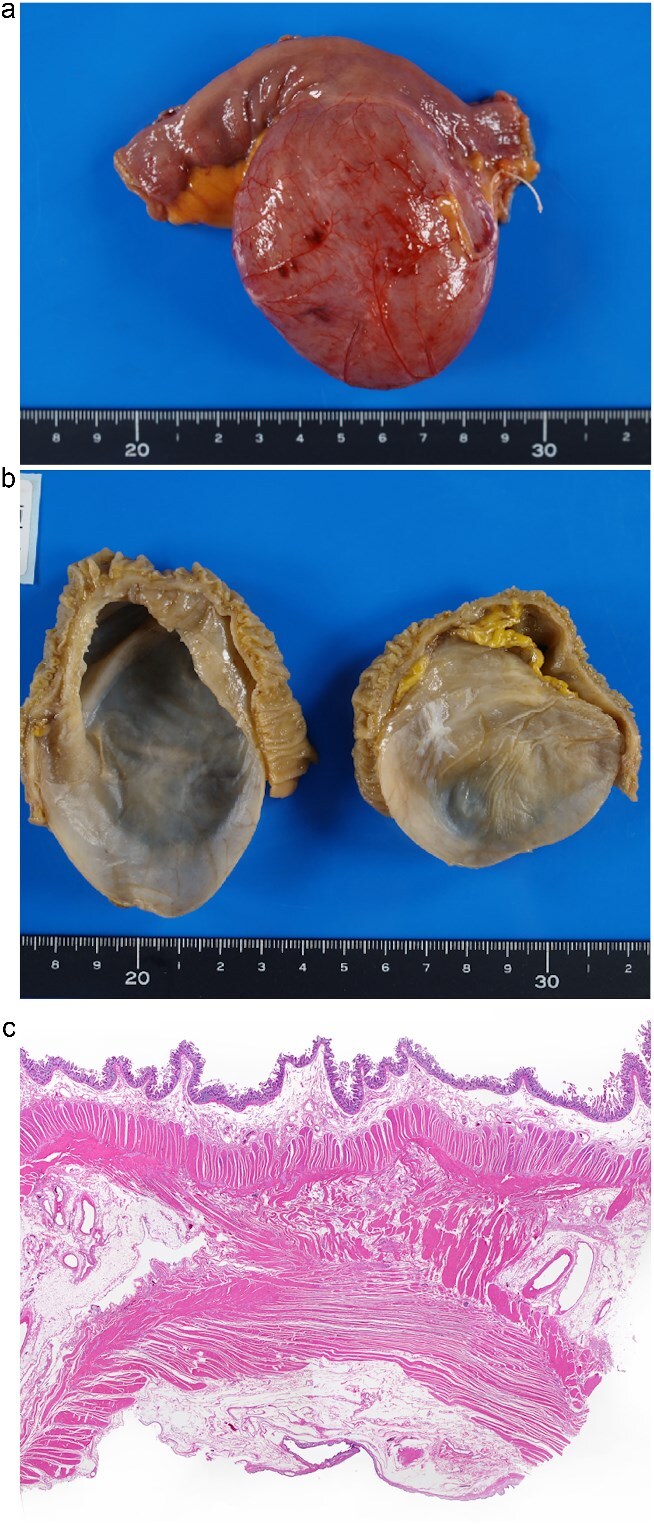
Macroscopic and histopathological findings of the resected specimen. (a) Gross specimen of the cystic lesion attached to the ileum. (b) Internal surface of the cystic lumen, appearing smooth and uniform. (c) Histological section stained with haematoxylin and eosin. The cyst wall consisted of columnar epithelium and a two-layered smooth muscle wall. There was no mucosal communication with the adjacent ileum, but partial sharing of the muscle layer was observed (H&E stain, magnification ×100).

Histopathological examination revealed a cyst lined by columnar epithelium and surrounded by a double-layered smooth muscle wall, consistent with alimentary tract duplication. The cyst had no mucosal communication with the ileal lumen, confirming the non-communicating cystic type (Fig. 3a–c). The patient’s postoperative course was uneventful, and she was discharged on postoperative day 7 without complications.

## Discussion

Alimentary tract duplication is a rare congenital anomaly typically diagnosed in infancy [1, 4]. Adult cases are rare and often discovered incidentally. The present case was diagnosed preoperatively owing to dynamic imaging findings including peristaltic motion and the characteristic layered wall, features highly suggestive of alimentary tract duplication.

Ultrasonography demonstrated a multilayered cystic wall and dynamic changes in shape over time, corresponding to the so-called ``gut signature'' [6]. MRI, particularly T2-weighted sequences, further confirmed peristaltic activity within the cyst and provided valuable information on its anatomical relationship with surrounding structures [7].

Detection of peristalsis on imaging, while rarely reported, provides strong evidence for the presence of functional smooth muscle and aids differentiation from other cystic lesions. Literature suggests that only ~11% of alimentary duplications are diagnosed preoperatively [9]. In this context, real-time ultrasonography and dynamic MRI can significantly enhance diagnostic confidence [8].

The differential diagnosis of cystic lesions in the abdomen, particularly in asymptomatic adults, is broad and includes mesenteric cysts, Meckel’s diverticulum, ovarian cysts, and enteric duplication cysts. Among these, only enteric duplications demonstrate peristalsis and the gut signature, making dynamic imaging a powerful diagnostic discriminator [5–7]. Furthermore, awareness of such rare pathologies is essential to avoid misdiagnosis and unnecessary interventions [4, 5].

Surgically, accurate preoperative identification of the lesion allowed for strategic planning of a minimally invasive approach. Single-incision laparoscopic surgery (SILS) offers superior cosmetic outcomes and potentially reduced postoperative pain compared to conventional laparoscopy [10]. In the present case, SILS was safely and effectively employed, illustrating its feasibility even in older adults. As laparoscopic techniques become more refined, their application to rare pathologies such as enteric duplications will likely expand [10].

Histologically, the correlation between imaging features and pathological confirmation of smooth muscle layers and columnar epithelium is noteworthy [6]. This strengthens the concept that imaging can reliably reflect underlying tissue architecture, thereby assisting in surgical planning and patient counseling [7].

In summary, this case underscores the diagnostic and therapeutic significance of dynamic ultrasonography and MRI in adult-onset alimentary tract duplication. Radiologic-pathologic correlation, combined with minimally invasive techniques, contributes to favourable outcomes in rare congenital anomalies presenting in adulthood.

## Supplementary Material

Supplementary_Video1_rjaf834

Supplementary_Video2_rjaf834
